# Low oxygen saturation and mortality in an adult cohort: the Tromsø study

**DOI:** 10.1186/s12890-015-0003-5

**Published:** 2015-02-12

**Authors:** Monica Linea Vold, Ulf Aasebø, Tom Wilsgaard, Hasse Melbye

**Affiliations:** Department of Respiratory Medicine, University Hospital of North Norway, 9038 Tromsø, Norway; Department of Community Medicine, University of Tromsø, Tromsø, Norway; Department of Clinical Medicine, University of Tromsø, Tromsø, Norway

## Abstract

**Background:**

Oxygen saturation has been shown in risk score models to predict mortality in emergency medicine. The aim of this study was to determine whether low oxygen saturation measured by a single-point measurement by pulse oximetry (SpO_2_) is associated with increased mortality in the general adult population.

**Methods:**

Pulse oximetry was performed in 5,152 participants in a cross-sectional survey in Tromsø, Norway, in 2001–2002 (“Tromsø 5”). Ten-year follow-up data for all-cause mortality and cause of death were obtained from the National Population and the Cause of Death Registries, respectively. Cause of death was grouped into four categories: cardiovascular disease, cancer except lung cancer, pulmonary disease, and others. SpO_2_ categories were assessed as predictors for all-cause mortality and death using Cox proportional-hazards regression models after correcting for age, sex, smoking history, body mass index (BMI), C-reactive protein level, self-reported diseases, respiratory symptoms, and spirometry results.

**Results:**

The mean age was 65.8 years, and 56% were women. During the follow-up, 1,046 (20.3%) participants died. The age- and sex-adjusted hazard ratios (HRs) (95% confidence intervals) for all-cause mortality were 1.99 (1.33–2.96) for SpO_2_ ≤ 92% and 1.36 (1.15–1.60) for SpO_2_ 93–95%, compared with SpO_2_ ≥ 96%. In the multivariable Cox proportional-hazards regression models that included self-reported diseases, respiratory symptoms, smoking history, BMI, and CRP levels as the explanatory variables, SpO_2_ remained a significant predictor of all-cause mortality. However, after including forced expiratory volume in 1 s percent predicted (FEV_1_% predicted), this association was no longer significant. Mortality caused by pulmonary diseases was significantly associated with SpO_2_ even when FEV_1_% predicted was included in the model.

**Conclusions:**

Low oxygen saturation was independently associated with increased all-cause mortality and mortality caused by pulmonary diseases. When FEV_1_% predicted was included in the analysis, the strength of the association weakened but was still statistically significant for mortality caused by pulmonary diseases.

## Background

Pulse oximeters are cheap and are used widely as non-invasive devices for estimating oxygen saturation (SpO_2_). Pulse oximetry is used extensively in clinical medicine to evaluate and monitor patients. Low oxygen saturation or hypoxemia is associated with conditions or diseases involving ventilation–perfusion mismatch in the lungs, hypoventilation, right-to-left shunts, reduced diffusion capacity, and reduced oxygen partial pressure in inspired air. There is no clear cut-off point for abnormal oxygen saturation, but SpO_2_ ≤ 95% is used in most adult studies. In materials for blood gas reference values, Crapo et al. reported a mean arterial oxygen saturation (SaO_2_) of 95.5–96.9% (standard deviation (SD) 0.4–1.4) [1]. In a more recent paper, the median SaO_2_ was 98.2% (range 96.6–99.5%) in the 20–39-year-old age group and 98.0% (range 95.1–99.7%) in the 40–76-year-old age group [2]. SaO_2_ decreased marginally with age by about 0.20% per decade. A resting SpO_2_ ≤ 95% has been found to predict oxygen desaturation during sleep, exercise, and air plane travel in chronic obstructive pulmonary disease (COPD) patients [3-5]. SpO_2_ ≤ 95% has also been identified as a risk factor for postoperative pulmonary complications [6]. The value of 96% seems a reasonable cut-off value.

An SpO_2_ cut-off value of ≤92% is used when screening for respiratory failure in COPD patients [7]. In emergency medicine, low SpO_2_ has been shown to be associated with increased mortality [8,9] and is included together with other vital signs when calculating the risk score for predicting prognosis [10-13]. Different risk-scoring models to predict mortality use different limits from <90 to ≤95% [10-14]. In lung diseases such as COPD, the partial pressure of oxygen (PaO_2_) is used most often in models to predict mortality [15]. Higher oxygen saturation has been shown in survivors [16,17], but neither SpO_2_ nor PaO_2_ was found to be a significant predictor when added to a validated multi-dimensional disease rating that included the body mass index (B), degree of airflow obstruction (O), dyspnoea (D), and exercise capacity (E) (BODE Index) in multivariable analysis [15].

There is limited information about low oxygen saturation and its association with mortality in the general population. In a previous study, we found that the most important predictors for low oxygen saturation in an adult population were increased body mass index (BMI) and reduced lung function, which was defined as decreased forced expiratory volume in 1 s percent predicted (FEV_1_% predicted) [18]. We also found that smoking history, dyspnoea, elevated haemoglobin concentration, age, and male sex predicted low oxygen saturation. FEV_1_% predicted is a predictor of mortality in both surveys of the general population [19] and COPD studies [15]. Low BMI has been associated with increased mortality both in epidemiological surveys [20,21] and COPD studies [22,23].

It is known that older age, male sex, smoking history (both current and former smoker), pack years (former smoker is often not significant when pack years are included) [19], and a history of cardiovascular disease (CVD), hypertension, or diabetes predict mortality in studies of the general adult population [24]. Biomarkers such as increased C-reactive protein (CRP) concentration have been found to predict mortality in both the general population [25] and patients with COPD [26].

The aim of this study was to examine whether a single-point measurement of a low SpO_2_ is associated with all-cause mortality and cause of death, especially death due to pulmonary diseases, in the general adult population after correcting for other established risk factors.

## Methods

### Subjects

**Figure 1:**
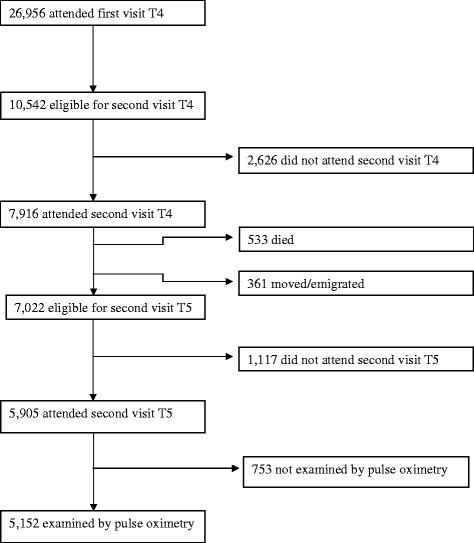
**Flow chart of participants from Tromsø 4 (T4) to Tromsø 5 (T5).**

The Tromsø Study comprises repeated cross-sectional population-based surveys, which were initiated in 1974 [27]. Tromsø is a university city in northern Norway where the population recently exceeded 70,000. Tromsø is situated at sea-level, and the oxygen partial pressure in inspired air is not reduced. The fifth Tromsø Study survey was performed in 2001–2002 and was conducted by the Department of Community Medicine, University of Tromsø, in co-operation with the National Health Screening Service. In the fourth survey, all inhabitants aged 55–74 years and 5–10% of the samples in the other age groups between 25 and 84 years were asked to take part in a second, more-extensive medical examination (77% agreed to participate). All participants from this second visit were invited to participate in the Tromsø 5 survey and were eligible for a second visit. In Tromsø 5, the first visit was attended by 8,130 subjects, which was 79% of those invited. At the second visit, 5,905 attended (84%), and SpO_2_ was measured by pulse oximetry in 5,152 participants (Figure 1). Lack of staff was the main reason why pulse oximetry and spirometry were not performed in 13% of the participants.

### Examinations

A questionnaire was mailed together with an invitation to participate in the study. The questionnaire included questions about the participant’s history of diseases, respiratory symptoms, and smoking habits. Participants who reported experiencing angina pectoris, myocardial infarction, or cerebral stroke were classified as having “self-reported CVD”. Participants who used antihypertensive drugs were classified as having “self-reported hypertension”. The examinations at the first visit included height and weight, and BMI (kg/m^2^) was calculated.

Pulse oximetry and spirometry were measured during the second visit. SpO_2_ values were measured with a digital handheld pulse oximeter (Onyx II, model 9550, Nonin Medical, Inc., Plymouth, MN, USA). The participants rested at least 15 minutes before the measurement. The highest of three measurements was recorded. The manufacturer’s testing has shown that only values between 70% and 100% are accurate to within ±2 digits, and values <70% were regarded as invalid.

Spirometry was performed using the Vmax Legacy 20 system (VIASYS Healthcare Respiratory Technologies, Yorba Linda, CA, USA). American Thoracic Society criteria [28] were followed. Norwegian reference values for pre-bronchodilatory spirometry [29] were used because reversibility testing was not performed. Calibration of the instrument was performed every morning and as the machine required. Three trained technicians conducted the spirometry. Current drug therapy was not interrupted before the test. Both pulse oximetry and spirometry were recorded in 5,131 individuals, and a valid FEV_1_% predicted was obtained in 4,988 of these participants.

On the same day, as part of the second examination, blood was drawn for measurement of the concentrations of haemoglobin [30] and CRP. CRP concentration was measured using a high-sensitivity immunoturbidimetric assay [31].

### Statistical analysis

Ten-year follow-up data for all-cause mortality were obtained from the National Population Register of Norway and causes of death from the National Cause of Death Registry. Subjects who emigrated were censored at the date of emigration. If subjects had not died or emigrated, they were censored at 10 years from the baseline. The causes of death were classified into four categories: CVD, cancer except lung cancer, pulmonary disease (including COPD, asthma, interstitial lung diseases, sequelae of tuberculosis, and lung cancer), and others. Continuous variables were categorized. We defined a low pulse oximetry value as an SpO_2_ ≤ 95%. SpO_2_ values were categorized into three groups: reduced, ≤92%; mildly reduced, 93–95%; and normal, ≥96%. Characteristics of the participants were categorized according to SpO_2_ and mortality status, and differences were assessed using the chi-square test.

Associations with all-cause mortality and mortality caused by pulmonary diseases were analysed by Cox proportional-hazards regression for SpO_2_, smoking history, self-reported respiratory symptoms and diseases, BMI, CRP concentration, and spirometry measures, and were adjusted for age and sex. The significant predictors of mortality at the 5% level were entered into multivariable Cox proportional-hazards regression models. Knowing that FEV_1_% predicted is associated with both SpO_2_ and mortality, models with and without FEV_1_% predicted included were applied. IBM SPSS statistical software version 20 (IBM, Armonk, NY, USA) was used.

The Regional Committee for Medical and Health Research Ethics in North Norway approved the Tromsø 5 survey. All participants gave written informed consent.

## Results

**Figure 2:**
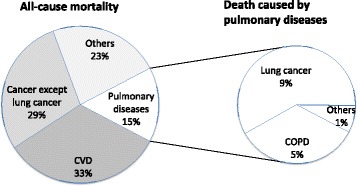
**Causes of death for the 1,046 deaths.**

SpO_2_ values were recorded for 5,152 people in Tromsø 5. Their mean age was 65.8 years (SD 9.5; range 32–89 years), and 2,887 (56%) were women. During the follow-up period from 2001–2002 until 2011–2012, 1,046 (20.3%) died: 346 (33.1%) died of CVD, 299 (28.6%) of cancer except lung cancer, 161 (15.4%) of pulmonary disease, and 240 (22.9%) of other diseases (Figure 2). The mean follow-up period was 9.2 years (SD 2.0). An SpO_2_ ≤ 95% was found in 11.5% of the population.

Table 1 shows the baseline characteristics grouped according to SpO_2_. Low SpO_2_ was significantly associated with older age, self-reported diseases and symptoms, smoking history, high BMI and CRP concentration, and low FEV_1_% predicted and FEV_1_/forced vital capacity (FVC)%. A high haemoglobin concentration was not significantly associated with low SpO_2_ and was not included in further analysis.

**Table 1:** **Baseline characteristics classified by arterial oxygen saturation (SpO**_**2**_ **) in 5,152 participants**

	**≤92%**		**93–95%**		**≥96%**		**p-value**
	**n**	**(%)**	**n**	**(%)**	**n**	**(%)**	
All		53	(1.0)	537	(10.4)	4,562	(88.5)
Sex							0.47
Female	34	(1.2)	297	(10.3)	2,556	(88.5)	
Male	19	(0.8)	240	(10.6)	2,006	(88.6)	
Age (years)							<0.001^*^
<60	1	(0.1)	57	(5.3)	1,027	(94.7)	
60–70	16	(0.7)	217	(10.2)	1,902	(89.1)	
≥70	36	(1.9)	263	(13.6)	1,633	(84.5)	
Self-reported diseases							
CVD							<0.001
No	39	(0.9)	410	(9.6)	3,814	(89.5)	
Yes	14	(1.6)	127	(14.3)	748	(84.1)	
Asthma							<0.001
No	37	(0.8)	448	(9.6)	4,197	(89.6)	
Yes	16	(3.4)	89	(18.9)	365	(77.7)	
COPD							<0.001
No	39	(0.8)	476	(9.8)	4,366	(89.4)	
Yes	14	(5.2)	61	(22.5)	196	(72.3)	
Diabetes							0.004
No	49	(1.0)	499	(10.1)	4,373	(88.9)	
Yes	4	(1.7)	38	(16.5)	189	(81.8)	
Hypertension							0.020
No	40	(1.0)	378	(9.7)	3,463	(89.2)	
Yes	13	(1.0)	159	(12.5)	1099	(86.5)	
Smoking history							0.001^*^
Never	16	(0.9)	141	(8.1)	1,581	(91.0)	
Former	21	(1.0)	241	(11.6)	1,819	(87.4)	
Current	16	(1.2)	155	(11.6)	1,162	(87.2)	
Self-reported symptoms							
Dyspnoea^#^							<0.001^*^
0	17	(0.6)	221	(8.0)	2,511	(91.3)	
1	17	(0.8)	244	(11.9)	1,785	(87.2)	
≥2	19	(5.3)	72	(20.2)	266	(74.5)	
Chronic cough with sputum							<0.001
No	44	(0.9)	460	(9.7)	4,261	(89.4)	
Yes	9	(2.3)	77	(19.9)	301	(77.8)	
BMI (kg/m^2^)							<0.001^*^
<18.5	1	(2.0)	6	(12.2)	42	(85.7)	
18.5-24.9	16	(1.0)	107	(6.4)	1,548	(92.6)	
25.0-29.9	21	(0.9)	239	(10.2)	2,082	(88.9)	
≥30.0	15	(1.4)	180	(17.0)	865	(81.6)	
CRP (mg/L)							<0.001
<5	36	(0.8)	421	(9.7)	3,871	(89.4)	
≥5	17	(2.3)	109	(14.7)	615	(83.0)	
Haemoglobin^§^							0.29
≤upper limit	48	(1.0)	469	(10.3)	4,052	(88.7)	
>upper limit	0	(0.0)	4	(21.1)	15	(78.9)	
FEV_1_% predicted							<0.001^*^
<50	16	(8.2)	66	(34.0)	112	(57.7)	
50–80	22	(1.3)	223	(13.3)	1,427	(85.3)	
≥80	13	(0.4)	227	(7.3)	2,882	(92.3)	
FEV_1_/FVC%							<0.001
<0.7	26	(2.4)	185	(16.8)	890	(80.8)	
≥0.7	25	(0.6)	335	(8.6)	3,551	(90.8)	

^*^Chi-square trend.

#Dyspnoea: 0, no dyspnoea; 1, dyspnoea walking rapidly on level ground or up a moderate slope; ≥2, dyspnoea walking slowly on level ground, washing or dressing, or at rest.

^§^Upper limits: women, 16.0 g/dL; men, 17.0 g/dL.

Definitions of abbreviations: SpO_2_, arterial oxygen saturation as measured by pulse oximetry; CVD, cardiovascular disease; COPD, chronic obstructive pulmonary disease; BMI, body mass index; CRP, C-reactive protein; FEV_1_, forced expiratory volume in 1 s; FVC, forced vital capacity.

Table 2 shows the characteristics according to mortality status. The group of participants who had died were more likely to have been older and male; to have smoked more; and to have had more self-reported diseases and respiratory symptoms, a lower BMI, FEV_1_% predicted, FEV_1_/FVC%, and SpO_2_, and a higher CRP concentration. The frequency of death due to pulmonary diseases increased by decreasing SpO_2_: 104 out of 4563 (2.3%) participants with baseline SpO_2_ > 96%, 45 out of 537 (8.4%) with SpO_2_ 93-95%, and 12 out of 53(22.6%) with SpO_2_ ≤ 92%, p < 0.001.

**Table 2:** **Baseline characteristics classified by 10-year mortality status in 5,152 participants**

	**Total (n)**	**Dead (n)**	**%**	**p-value**
All	5,152	1,046	(20.3)	
Sex				<0.001
Female	2,887	459	(15.9)	
Male	2,265	587	(25.9)	
Age (years)				<0.001^*^
<60	1,085	43	(4.0)	
60-70	2,135	274	(12.8)	
≥70	1,932	729	(37.7)	
Self-reported diseases				
CVD				<0.001
No	4,263	742	(17.4)	
Yes	889	304	(34.2)	
Asthma				<0.001
No	4,682	914	(19.5)	
Yes	470	132	(28.1)	
COPD				<0.001
No	4,881	950	(19.5)	
Yes	271	96	(35.4)	
Diabetes				<0.001
No	4,921	959	(19.5)	
Yes	231	87	(37.7)	
Hypertension				<0.001
No	3,881	697	(18.0)	
Yes	1271	349	(27.5)	
Smoking history				<0.001^*^
Never	1,736	269	(15.5)	
Former	2,083	464	(22.3)	
Current	1,333	313	(23.5)	
Self-reported symptoms				
Dyspnoea^#^				<0.001^*^
0	2,749	486	(17.7)	
1	2,046	437	(21.4)	
≥2	357	123	(34.5)	
Chronic cough with sputum				
No	4,765	920	(19.3)	<0.001
Yes	387	126	(32.6)	
BMI (kg/m^2^)				<0.001^*^
<18.5	49	22	(44.9)	
18.5-24.9	1,671	367	(22.0)	
25.0-29.9	2,342	451	(19.3)	
≥30.0	1,060	193	(18.2)	
CRP (mg/L)				<0.001
<5	4,328	808	(18.7)	
≥5	741	213	(28.7)	
FEV_1_% predicted				<0.001^*^
<50	194	99	(51.0)	
50–80	1,672	434	(26.0)	
≥80	3,122	456	(14.6)	
FEV_1_/FVC%				<0.001
<70	1,101	386	(35.1)	
≥70	3,911	612	(15.6)	
SpO_2_ (%)				<0.001^*^
≤92	53	25	(47.2)	
93–95	537	163	(30.4)	
≥96	4,562	858	(18.8)	

^*^Chi-square trend.

^#^Dyspnoea: 0, no dyspnoea; 1, dyspnoea walking rapidly on level ground or up a moderate slope; ≥2, dyspnoea walking slowly on level ground, washing or dressing, or at rest.

Definitions of abbreviations: CVD, cardiovascular disease; COPD, chronic obstructive pulmonary disease; BMI, body mass index; CRP, C-reactive protein; FEV_1_, forced expiratory volume in 1 s; FVC, forced vital capacity; SpO_2_, arterial oxygen saturation as measured by pulse oximetry.

**Figure 3:**
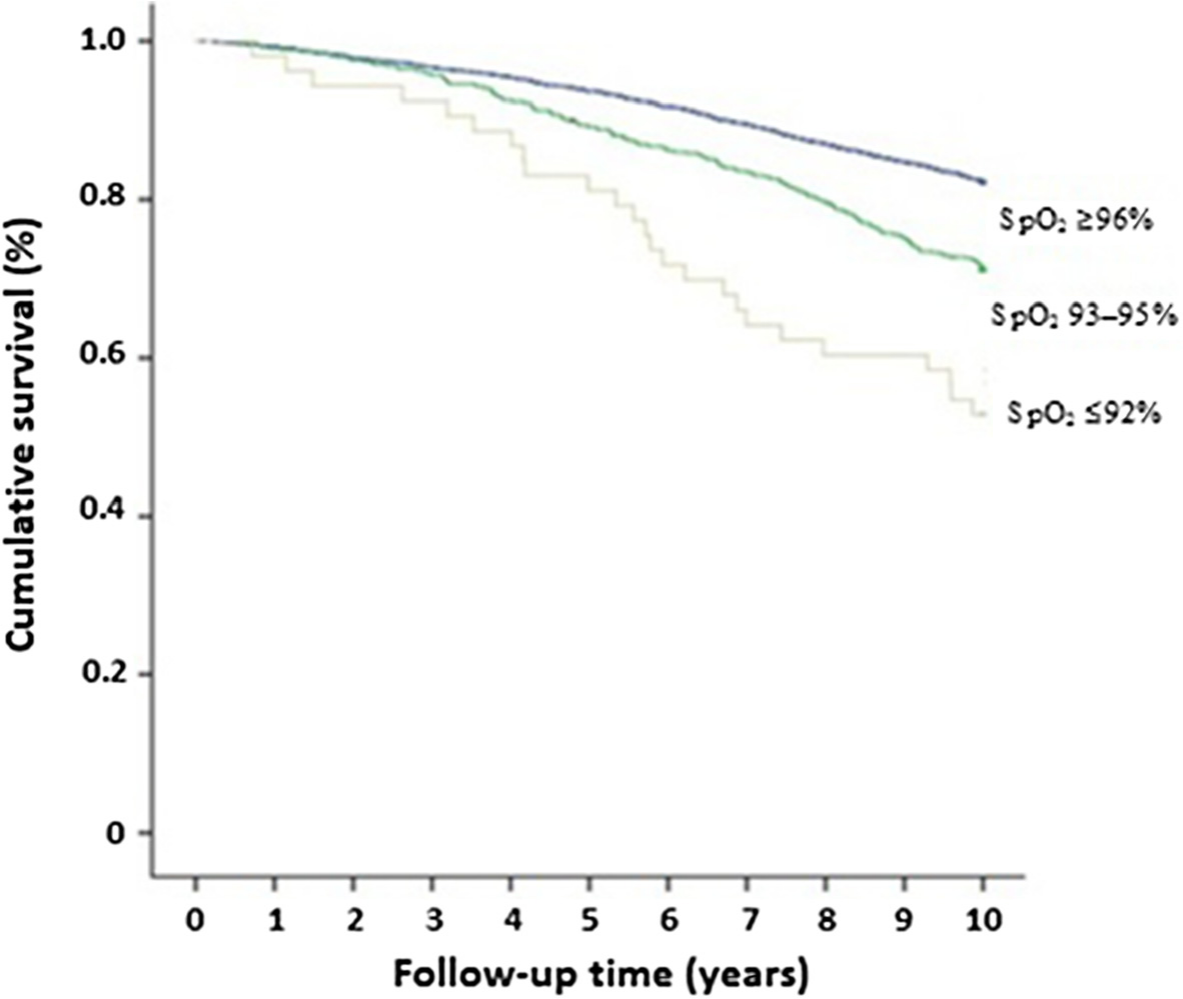
**Kaplan–Meier survival curves for different levels of oxygen saturation (SpO**_**2**_ **).**

Figure 3 shows the Kaplan–Meier survival curve for the different levels of SpO_2_. After adjusting for age and sex in the Cox proportional-hazards regression, the following factors were significantly associated with all-cause mortality and mortality caused by pulmonary diseases: lower SpO_2_, FEV_1_% predicted, FEV_1_/FVC%, and BMI; higher CRP concentration; smoking history; and self-reported diseases and respiratory symptoms (Table 3). The highest HRs for all-cause mortality were found for FEV_1_% predicted <50, current smoking, history of diabetes, and SpO_2_ ≤ 92% (3.07, 2.11, 2.08, and 1.99, respectively). For pulmonary diseases, the highest HRs were found for FEV_1_% predicted <50, current smoking, and SpO_2_ ≤ 92% (16.35, 14.21, and 9.12, respectively) (Table 4).

**Table 3:** **Hazard ratios for 10-year all-cause mortality in 3 different models** ^*^

	**Model 1**			**Model 2**			**Model 3**		
	**HR**	**(95% CI)**	**p-value**	**HR**	**(95% CI)**	**p-value**	**HR**	**(95% CI)**	**p-value**
Age (years)				1.13	(1.12–1.14)	<0.001	1.12	(1.11–1.14)	<0.001
Sex									
Female					1	(reference)		1	(reference)
Male				1.70	(1.49–1.95)	<0.001	1.66	(1.44–1.91)	<0.001
Self-reported diseases									
CVD	1.34	(1.17–1.54)	<0.001	1.18	(1.01–1.37)	0.033	1.18	(1.01–1.37)	0.035
Asthma	1.33	(1.10–1.59)	0.003	1.03	(0.84–1.25)	0.81	0.92	(0.74–1.13)	0.41
COPD	1.75	(1.42–2.16)	<0.001	1.29	(1.02–1.64)	0.036	1.13	(0.88–1.44)	0.35
Diabetes	2.08	(1.67–2.59)	<0.001	2.09	(1.66–2.63)	<0.001	1.88	(1.48–2.40)	<0.001
Hypertension	1.20	(1.05–1.36)	0.006	1.24	(1.08–1.43)	0.003	1.24	(1.07–1.43)	0.004
Smoking history									
Never	1	(reference)		1	(reference)		1	(reference)	
Former	1.29	(1.10–1.52)	0.002	1.24	(1.05–1.46)	0.010	1.16	(0.98–1.38)	0.082
Current	2.11	(1.78–2.49)	<0.001	1.89	(1.58–2.26)	<0.001	1.68	(1.39–2.03)	<0.001
Self-reported symptoms									
Dyspnoea^€^									
0	1	(reference)		1	(reference)		1	(reference)	
1	1.19	(1.05–1.36)	0.007	1.06	(0.92–1.22)	0.42	1.02	(0.89–1.18)	0.76
≥2	1.71	(1.40–2.08)	<0.001	1.32	(1.06–1.64)	0.015	1.15	(0.92–1.46)	0.23
Chronic cough with sputum									
Yes	1.71	(1.42–2.07)	<0.001	1.28	(1.04–1.57)	0.018	1.25	(1.01–1.54)	0.042
BMI (kg/m^2^)									
<18.5	2.08	(1.35–3.20)	0.001	1.99	(1.27–3.11)	0.003	1.76	(1.08–2.84)	0.022
18.5–24.9	1	(reference)		1	(reference)		1	(reference)	
25.0–29.9	0.85	(0.74–0.97)	0.018	0.82	(0.71–0.95)	0.008	0.84	(0.73–0.98)	0.024
≥30.0	0.80	(0.68–0.96)	0.015	0.68	(0.56–0.82)	<0.001	0.71	(0.58–0.87)	0.001
CRP (mg/L)									
<5	1	(reference)		1	(reference)		1	(reference)	
≥5	1.57	(1.35–1.82)	<0.001	1.39	(1.19–1.63)	<0.001	1.36	(1.16–1.60)	<0.001
FEV_1_% predicted									
<50	3.07	(2.46–3.81)	<0.001				1.99	(1.51–2.61)	<0.001
50–80	1.54	(1.35–1.76)	<0.001				1.29	(1.11–1.49)	0.001
≥80	1	(reference)					1	(reference)	
FEV_1_/FVC%									
<0.7	1.69	(1.49–1.93)	<0.001				1.11	(0.95–1.31)	0.20
≥0.7	1	(reference)					1	(reference)	
SpO_2_ (%)									
≤92	1.99	(1.33–2.96)	0.001	1.73	(1.15–2.60)	0.008	1.42	(0.92–2.18)	0.11
93–95	1.36	(1.15–1.60)	<0.001	1.27	(1.06–1.51)	0.009	1.13	(0.94–1.36)	0.19
≥96	1	(reference)		1	(reference)		1	(reference)	

^*^Adjusted for age and sex in Model 1 and for all listed variables in the other two models.

^€^Dyspnoea: 0, no dyspnoea, 1, dyspnoea while walking rapidly on level ground or up a moderate slope, ≥2, dyspnoea while walking slowly on level ground, washing or dressing, or at rest.

Definition of abbreviations: HR, hazard ratio; CI, confidence interval; CVD, cardiovascular disease; COPD, chronic obstructive pulmonary disease; BMI, body mass index; CRP, C-reactive protein; FEV_1_, forced expiratory volume in 1 s; FVC, forced vital capacity; SpO_2_, arterial oxygen saturation as measured by pulse oximetry.

**Table 4:** **Hazard ratios for 10-year mortality due to pulmonary diseases** ^*^ **in 3 different models** ^**€**^

	**Model 1**			**Model 2**			**Model 3**		
	**HR**	**(95% CI)**	**p-value**	**HR**	**(95% CI)**	**p-value**	**HR**	**(95% CI)**	**p-value**
Age (years)				1.10	(1.07–1.12)	<0.001	1.08	(1.05–1.11)	<0.001
Sex									
Female				1	(reference)			1	(reference)
Male				2.12	(1.50–2.98)	<0.001	1.95	(1.37–2.79)	<0.001
Self-reported diseases									
CVD	1.16	(0.81–1.67)	0.42	1.21	(0.81–1.80)	0.36	1.28	(0.85–1.93)	0.23
Asthma	3.64	(2.57–5.16)	<0.001	1.96	(1.32–2.92)	0.001	1.53	(1.02–2.31)	0.042
COPD	5.18	(3.57–7.52)	<0.001	1.72	(1.10–2.69)	0.017	1.31	(0.83–2.06)	0.26
Diabetes	1.73	(0.94–3.19)	0.081	1.91	(1.01–3.64)	0.048	1.50	(0.76–2.98)	0.24
Hypertension	0.65	(0.44–0.95)	0.027	0.75	(0.49–1.14)	0.18	0.74	(0.48–1.12)	0.15
Smoking history									
Never	1	(reference)		1	(reference)			1	(reference)
Former	4.74	(2.41–9.34)	<0.001	3.78	(1.91–7.48)	<0.001	3.08	(1.55–6.12)	0.001
Current	14.21	(7.32–27.59)	<0.001	9.26	(4.69–18.28)	<0.001	6.35	(3.17–12.71)	<0.001
Self-reported symptoms									
Dyspnoea^#^									
0	1	(reference)		1	(reference)		1	(reference)	
1	1.64	(1.17–2.31)	0.005	1.09	(0.75–1.58)	0.65	0.99	(0.67–1.45)	0.94
≥2	3.39	(2.16–5.33)	<0.001	1.31	(0.77–2.24)	0.31	1.12	(0.64–1.94)	0.70
Chronic cough with sputum									
Yes	1.85	(1.24–2.77)	0.003	1.86	(1.25–2.79)	0.002	1.63	(1.07–2.48)	0.022
BMI (kg/m^2^)									
<18.5	2.92	(1.17–7.30)	0.022	1.75	(0.63–4.88)	0.29	0.83	(0.20–3.46)	0.80
18.5-24.9	1	(reference)		1	(reference)		1	(reference)	
25.0-29.9	0.62	(0.44–0.88)	0.007	0.67	(0.47–0.96)	0.031	0.73	(0.50–1.06)	0.09
≥30.0	0.66	(0.42–1.03)	0.064	0.67	(0.41–1.08)	0.099	0.79	(0.49–1.30)	0.36
CRP (mg/L)									
<5	1	(reference)		1	(reference)		1	(reference)	
≥5	1.90	(1.32–2.74)	0.001	1.13	(0.77–1.68)	0.53	1.06	(0.71–1.58)	0.78
FEV_1_% predicted									
<50	16.35	(10.51–25.43)	<0.001				3.74	(2.07–6.77)	<0.001
50–80	3.11	(2.10–4.60)	<0.001				1.73	(1.11–2.68)	0.015
≥80	1	(reference)					1	(reference)	
FEV_1_/FVC%									
<0.7	5.18	(3.70–7.25)	<0.001				1.64	(1.08–2.51)	0.022
≥0.7	1	(reference)					1	(reference)	
SpO_2_ (%)									
≤92	9.12	(4.99–16.67)	<0.001	5.19	(2.70–9.99)	<0.001	3.17	(1.53–6.56)	0.002
93–95	3.32	(2.33–4.71)	<0.001	2.54	(1.75–3.68)	<0.001	1.97	(1.33–2.92)	0.001
≥96	1	(reference)		1	(reference)		1	(reference)	

^*^Pulmonary diseases: including COPD, asthma, interstitial lung disease, sequelae of tuberculosis, lung cancer.

^€^Adjusted for age and sex in Model 1 and for all listed variables in the other two models.

^#^Dyspnoea: 0, no dyspnoea; 1, dyspnoea while walking rapidly on level ground or up a moderate slope, ≥2, dyspnoea walking slowly on level ground, washing or dressing, or at rest.

Definition of abbreviations: HR, hazard ratio; CI, confidence interval; CVD, cardiovascular disease; COPD, chronic obstructive pulmonary disease; BMI, body mass index; CRP, C-reactive protein; FEV_1_, forced expiratory volume in 1 s; FVC, forced vital capacity; SpO_2_, arterial oxygen saturation as measured by pulse oximetry.

A multivariable Cox proportional-hazards regression model for all-cause mortality that included all the variables except spirometry values produced HRs of 1.73 (95% confidence interval (CI) 1.15–2.60) and 1.27 (95% CI 1.06–1.51) for an SpO_2_ ≤ 92% and 93–95%, respectively. However, adding FEV_1_% predicted as an explanatory variable in the model decreased the HRs of SpO_2_ significantly, and although the association indicated a trend, it was not significant (Table 3).

Using the same models with mortality caused by pulmonary diseases as the outcome (Table 4), SpO_2_ was a significant variable, even when FEV_1_% predicted was included. The HRs for SpO_2_ ≤ 92% and 93–95% were 3.17 (95% CI 1.53–6.56) and 1.97 (95% CI 1.33–2.92), respectively. Examining the HR of low SpO_2_ for any other cause of death showed no significant associations except for heart failure (20 deaths), which occurred in a subgroup of those who had died from CVD. The HRs for death caused by heart failure was also significantly increased when FEV_1_% predicted was included in the model. FEV_1_% predicted was significantly associated with mortality caused by CVD, cancer except lung cancer, and pulmonary diseases but not with other diseases.

FEV_1_/FVC% was not significantly associated with all-cause mortality when included as a dichotomous variable (threshold of <0.7) or as a continuous variable in the multivariable model that included FEV_1_% predicted. FEV_1_/FVC% was a significant independent predictor of death caused by pulmonary diseases.

Cox proportional-hazards regression was also performed with the independent variables as continuous variables excluded by backward stepwise elimination. Only predictors with p < 0.05 were kept in the final model. With all the variables in the model, the HR per % SpO_2_ was 0.96 (95% CI 0.92–1.00; p = 0.026) and the HR per % FEV_1_% predicted was 0.99 (95% CI 0.98–0.99; p < 0.001).

## Discussion

In this study, we found that low oxygen saturation, defined as SpO_2_ ≤ 95% measured by a single-point measurement with pulse oximetry, was associated with increased all-cause mortality and mortality caused by pulmonary diseases. This has not been described previously in population studies. This association remained significant after adjusting for sex, age, history of smoking, self-reported diseases and respiratory symptoms, BMI, and CRP concentration. When including FEV_1_% predicted as a covariate, the HR for low SpO_2_ remained significant for pulmonary diseases but was no longer significant for all-cause mortality. The severity of COPD and pulmonary diseases, and death by respiratory failure seem to be predicted by low SpO_2_ in addition to spirometry in the general population.

There are probably several explanations as to why oxygen saturation is associated with mortality. Low SpO_2_ is a marker of cardiopulmonary diseases, which are among the leading causes of death in this population. Thirty-three per cent of deaths were caused by CVD, and 14% of deaths were caused by lung cancer and COPD. CVD predisposes a person to heart failure, which may affect pulmonary function and cause low SpO_2_. Even though SpO_2_ was not a significant predictor of death caused by CVD, we found a significant association with death caused by heart failure even when spirometry was included in the multivariable analysis. It is not surprising that low lung function, as measured by SpO_2_ and spirometry, is associated with death caused by pulmonary diseases. Lung cancer is associated with other respiratory diseases [32]. Severe respiratory disease in people with lung cancer limits the treatment modalities, among other surgery, and hence lower survival [33]. SpO_2_ has been shown to be a predictor of survival in lung cancer [34]. Spirometry has limitations in assessing the severity of pulmonary diseases, especially in the presence of reduced diffusion capacity as occurs in emphysema and interstitial lung disease. Therefore, SpO_2_ may be an independent risk factor when the results of other lung function tests, such as the 6 min walk test or diffusing capacity/transfer factor of the lung for carbon monoxide, are not available.

### Comparison with previous studies

In a recently published study [18], we reported a prevalence of 6.3% for SpO_2_ ≤ 95% in Tromsø 6, which was lower than the 11.5% found in this study from Tromsø 5. The main reason for this difference is probably that a higher percentage was smokers in Tromsø 5 than in Tromsø 6 (25.9% and 18.0%, respectively). There was also a higher mean age in Tromsø 5: 65.8 years (SD 9.5) compared with 63.6 (SD 9.2) in Tromsø 6. The most important predictors of low SpO_2_ in Tromsø 6, BMI and FEV_1_% predicted, were significantly associated with mortality in a multivariable model in the present study. However, survival was not significantly lower for people with a higher BMI even though a higher BMI level was associated with low SpO_2_. Obesity is associated with sleep apnoea [35], obesity hypoventilation [36], diabetes, hypertension, and CVD [37]. Sleep apnoea is associated with lower daytime PaO_2_ even in people with normal spirometry values [38]. After correcting for these factors, obesity itself is not associated with higher mortality. In fact, for all-cause mortality it seems to have a protective effect. Although overweight and obesity may lead to decreased oxygen saturation, the risk of premature death seems not to be increased as long as the lung function is normal and other comorbidities are adjusted for. When including other comorbidities such as CVD, hypertension, and diabetes, other studies have found that obesity, when not very severe, does not increase mortality [39-41].

FEV_1_/FVC% was not observed to be significantly associated with all-cause mortality in the multivariable analysis. Both restrictive and obstructive airway diseases have been associated with increased mortality in previous studies [19,42], and both moderate to very severe COPD and restrictive lung diseases involve reduced FEV_1_% predicted. For the participants with an FEV_1_% predicted of <50%, almost 90% had an FEV_1_/FVC% <70, suggesting that the low oxygen saturation observed in this group was probably caused by COPD.

Male sex is associated with a shorter life expectancy than female sex. More men are former smokers and they tend to smoke more pack-years than women, which may explain some of the differences in life expectancy. The prevalence of CVD is higher in men. Similar findings have been found in another study [42].

Contrary to our previous study, we did in the current study not find that increased haemoglobin concentration was significantly associated with low SpO_2_. Few participants had a high haemoglobin concentration, and this value was missing in 11% of the participants. This might explain the lack of association in this study.

In a recent study, Smith et al. [43] reported increased mortality rates in hospitalized patients with an SpO_2_ < 96%. Increased mortality has also been found in emergency care patients with a low SpO_2_ [8,9]. SpO_2_ may be a good predictor of mortality in situations where spirometry is not available and in populations with a higher frequency of low SpO_2_, especially when used as part of a risk-scoring system.

### Strengths and weaknesses

This study was based on a single-point measurement of SpO_2_. We have not checked the reproducibility, but we know that the group with the lowest SpO_2_ (≤92%) in the follow-up examinations also showed consistently low values for SaO_2_ in blood gas analysis. Oxygen saturation can vary during the day, especially during activity and at night in people with a pulmonary disease such as COPD [44]. Baseline SpO_2_ (at rest) has been shown to predict oxygen desaturation during activity [3] and at night [4]. SpO_2_ can also be in the normal range even though FEV_1_% predicted is <50%.

The measurement of SpO_2_ could be a limitation because the accuracy of the device is ±2 digits. We tried to compensate for this possible confounding factor by using the highest of three measurements and categorizing the participants into groups.

The group with SpO_2_ ≤ 92% in this population was small and comprised only 1.0% of the entire population. One reason may be that people with the lowest values were too sick to participate. We might have found a stronger association with SpO_2_ in groups of patients with diseases such as COPD because such groups have a higher frequency of low SpO_2_.

The participation rate was lower in the oldest age group and in the youngest men. This might have affected our results by missing the sickest (oldest) and healthiest (youngest) groups.

We did not measure post-bronchodilator spirometry. A previous study has shown that this is probably not necessary when mortality is evaluated in population studies [42].

Recall bias and misclassification errors are major concerns when using questionnaires. A stronger association between smoking and mortality may have been observed if more valid data on pack-years had been obtained.

Measuring oxygen saturation by pulse oximetry has important limitations [45], especially when measuring values at the lower levels. Saturation may be overestimated in heavy smokers because high carboxyhaemoglobin levels may cause overestimation of the true SpO_2_. To validate the data for a particular device, future studies could include gas analysis in a subsample for comparison.

The cause of death may be uncertain or wrong in many instances because only a small percentage has an autopsy done (10–12% in Norway). Among the participants who died during this study, 36.9% had an FEV_1_/FVC% <70, and 9.2% reported having COPD. COPD as the main diagnosis or as one of the comorbidities was reported by only 6.5%.

## Conclusions

We observed that lower values from pulse oximetry were associated with increased all-cause mortality in the general adult population. This was probably because of the strong association with death caused by pulmonary diseases. The association was weakened and no longer statistically significant after adjusting for FEV_1_% predicted but remained significant for death caused by pulmonary diseases. Pulse oximetry is easy and safe to perform, and may be particularly useful in risk assessment when spirometry is not an option and when added to spirometry for assessing the risk of death because of pulmonary disease. Low pulse oximetry values found in a patient should warrant further examination.
